# Fabrication and Characterization of PZT Fibered-Epitaxial Thin Film on Si for Piezoelectric Micromachined Ultrasound Transducer

**DOI:** 10.3390/mi9090455

**Published:** 2018-09-11

**Authors:** Pham Ngoc Thao, Shinya Yoshida, Shuji Tanaka

**Affiliations:** Department of Robotics, Division of Mechanical Engineering, Tohoku University, Sendai 980-8579, Japan; s-yoshida@mems.mech.tohoku.ac.jp (S.Y.); tanaka@mems.mech.tohoku.ac.jp (S.T.)

**Keywords:** piezoelectric materials, epitaxial thin film, micromachined ultrasound transducer (pMUT)

## Abstract

This paper presents a fibered-epitaxial lead zirconate titanate (PZT) thin film with intermediate features between the monocrystalline and polycrystalline thin films for piezoelectric micromachined ultrasound transducer (pMUT). The grain boundaries confirmed by scanning electron microscopy, but it still maintained the in-plane epitaxial relationship found by X-ray diffraction analyses. The dielectric constant (*ε_r_*_33_ = 500) was relatively high compared to those of the monocrystalline thin films, but was lower than those of conventional polycrystalline thin films near the morphotropic phase boundary composition. The fundamental characterizations were evaluated through the operation tests of the prototyped pMUT with the fibered-epitaxial thin film. As a result, its piezoelectric coefficient without poling treatment was estimated to be *e*_31,*f*_ = −10–−11 C/m^2^, and thus reasonably high compared to polycrystalline thin films. An appropriate poling treatment increased *e*_31,*f*_ and decreased *ε_r_*_33_. In addition, this unique film was demonstrated to be mechanically tougher than the monocrystalline thin film. It has the potential ability to become a well-balanced piezoelectric film with both high signal-to-noise ratio and mechanical toughness for pMUT.

## 1. Introduction

A piezoelectric micromachined ultrasound transducer (pMUT) based on the flexural vibration of a membrane has been developed for various applications such as ultrasonic diagnostic, nondestructive evaluation of structures, proximity sensing and fingerprint identification [1,2,3,4,5,6,7,8]. Thanks to its small size, light weight, ultra-low power consumption and wafer-level mass-producibility, the pMUT is expected to be a large-volume microelectromechanical systems (MEMS) product that is widely used in mobile phones, toys, robots and so on.

The pMUT senses and transmits an acoustic wave utilizing the positive piezoelectric and inverse piezoelectric effects of a piezoelectric thin film, respectively. Normally, a movable membrane in the pMUT consists of a stacked structure with piezoelectric, conductive and passive elastic layers. In transmission mode, the membrane is vertically deflected by a stress generated in the piezoelectric thin film by applying a driving voltage, and thus transmits an ultrasonic wave by spring-back motion. For this mode, the transverse piezoelectric constant (*e*_31,*f*_) of the piezoelectric thin film is important. As *e*_31,*f*_ increases, the sound pressure becomes larger at the same driving voltage, or the required driving voltage becomes lower to obtain the same pressure level. In sense mode, the membrane is deflected by receiving an acoustic wave. As a result, a voltage output is generated in the inverse manner to the transmission mode. For this mode, a piezoelectric output constant, *g* constant (*e*_31,*f*_/*ε_r_*_33_*ε*_0_), is important, where *ε_r_*_33_ and *ε*_0_ correspond to the relative dielectric constant and dielectric constant of vacuum, respectively. Considering both modes, the figure of merit (FOM) for pMUTs is expressed as (*e*_31,*f*_)^2^/*ε_r_*_33_*ε*_0_ [2,9]. This expression shows that the piezoelectric thin film with a high piezoelectric constant and a low dielectric constant is desired to improve the performance of pMUTs.

Recently, an AlN-based pMUT has been successfully developed because an AlN thin film is available in MEMS foundry services [10,11]. However, a large driving voltage, e.g., a few dozen volts, is needed to generate a sound pressure large enough for its applications because the piezoelectricity of AlN is poor. The *g* constant is relatively high owing to its relative dielectric constant *ε_r_*_33_ as small as 10, which is theoretically advantageous. In practice, however, a too-small dielectric constant often leads to high susceptibility to parasitic capacitances in the device. A lead zirconate titanate (PZT) polycrystalline thin film has been also considered as a transducer material for long time. By contrast to AlN, it well works as a transmitter due to its large piezoelectricity, while the sensing performance is limited due to its large dielectric constants and the resultant small *g* constant. For example, the dielectric constants of PZT polycrystals near the morphotropic phase boundary (MPB) composition were 800–1800 [12,13,14,15,16,17,18]. Therefore, a trade-off relationship between piezoelectricity and the dielectric constant generally exists for piezoelectric materials, which results in limiting the FOM defined as (*e*_31,*f*_)^2^/*ε_r_*_33_*ε*_0_.

In order to break this relationship, we developed a highly *c*-axis-oriented monocrystalline thin film of PZT family near MPB composition [19,20]. This monocrystalline thin film exhibits large piezoelectricity (*e*_31,*f*_ = −10–−14 C/m^2^) and a small dielectric constant (*ε_r_*_33_ = 200–300). The high *c*-axis orientation and elimination of the grain boundary suppress the extrinsic factors such as domain motion and charge traps, increasing the dielectric constant [21,22]. This PZT monocrystalline thin film promises to create a high performance pMUT, considering the FOM [2,20].

One of the predicted weak points for the monocrystalline thin film is the mechanical toughness. The monocrystalline material is usually brittle and weak against tensile stress along specific crystal directions. The *c*-axis orientated PZT monocrystalline thin film normally stores a large tensile stress, which is caused by the large difference of the coefficients of thermal expansion between PZT and Si [19]. If a crack incidentally generates at a stressed-concentrated point in the film during operation, it will easily propagate along the crystal directions and break the device. From the above discussion, an ideal PZT thin film for pMUTs should have a polycrystalline structure but still show large *e*_31,*f*_ and small *ε_r_*_33_, like the monocrystalline thin film. Such an ambivalent material has never been reported.

In this study, we have focused attention on a PZT partially epitaxial thin film with a grain boundary in the in-plane direction as a candidate of such an ambivalent material. This film is called a fibered-epitaxial thin film in this paper. Thus far, although similar epitaxial thin films with three variants in the in-plane direction were fabricated by metal–organic chemical vapor deposition [23,24,25], nobody has investigated the piezoelectricity and applicability to MEMS. The thicknesses were less than 300 nm, which is too small for pMUT applications. We fabricated the fibered-epitaxial thin film on Si by sputter deposition, and investigated the fundamental properties. Its applicability to pMUT was then investigated through the operation experiment.

## 2. Materials and Methods

### 2.1. Sputter Deposition of Lead Zirconate Titanate (PZT) Fibered-Epitaxial Thin Film 

At first, oxide buffer layers of SrRuO_3_/LaSrCoO_3_/CeO_2_/YSZ were formed by pulsed laser deposition on a silicon-on-insulator (SOI) substrate. The electrical resistivity of SrRuO_3_ (SRO) is approximately 300 µΩ·m [26]. This resistivity is relatively low, enough to be utilized as the material not only to enhance the epitaxial growth of PZT but also for the bottom electrode. The detailed deposition condition is described elsewhere [19]. Then, Pb(Zr_0.5_,Ti_0.5_)O_3_ was deposited on the substrate at 0.5 Pa of the mixture of Ar and O_2_ gas by magnetron sputtering followed by fast cooling [19,20]. The target was prepared at the composition of Pb(Zr_0.5_,Ti_0.5_)O_3_ with an excess 10 mol% PbO to compensating the loss of the Pb component due to the vaporization in the sputtering process. The PZT monocrystalline thin film was obtained at 550–600 °C as the deposition temperature, while the fibered-epitaxial thin film was formed at a relatively lower deposition temperature of 500–550 °C to intentionally reduce the monocrystallinity.

### 2.2. Fabrication of pMUT Linear Array with Partially-Etched PZT Thin Film

**Figure 1:**
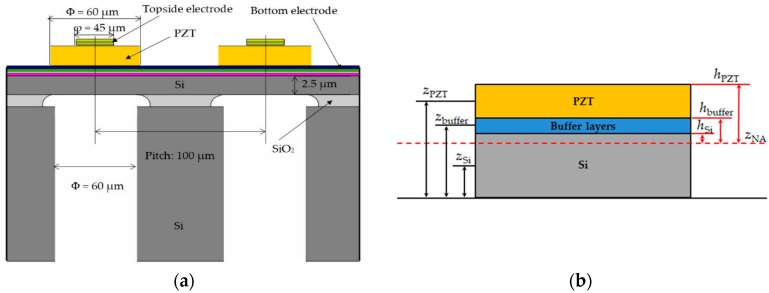
(**a**) Schematic illustration of the piezoelectric micromachined ultrasound transducer (pMUT) linear array with partially-etched lead zirconate titanate (PZT) thin film; (**b**) Simplified structure to calculate flexural rigidity.

A pMUT linear array based on the PZT fibered-epitaxial thin film was designed and fabricated to evaluate the fundamental properties. The schematic illustration is shown in Figure 1a. In this study, the diameter of the membrane was set to 60 μm. The thickness of the PZT thin film, a buffer layer and a Si elastic plate were 1.7 μm, 150 nm, 2.5 μm, respectively. The fundamental resonant frequency *f*_0_ for a unimorph pMUT was estimated to be 9 MHz from the following formula

(1) $$f_{0}=\frac{\lambda_{01}^{2}}{2\pi a^{2}}\sqrt{\frac{D}{\rho t}}$$

where *t*, *a*, *ρ*, *D* and *λ*_01_ correspond to the total thickness, radius, density, flexural rigidity of the membrane, and vibration mode eigenvalue, respectively.

This pMUT structure was assumed to be composed of three layers, namely the PZT thin film, buffer layer and Si elastic plate. The neutral axis position (*z*_NA_) should exist in the elastic plate or underlayer of the piezoelectric thin film normally, which is calculated as [27]

(2) $$z_{\mathrm{NA}}=\frac{\sum_{n=1}^{3}t_{n}z_{n}E_{n}^{\prime }}{\sum_{n=1}^{3}t_{n}E_{n}^{\prime }}$$

where *n* is the layer index (Si, buffer layer, PZT), *z_n_* is the distance between each layer’s middle plane and the bottom of the stack, as shown in Figure 1b. *t_n_* is the thickness of each layer.

The flexural rigidity, *D*, is defined as [28]

(3) $$D=\frac{1}{3}\sum_{n=1}^{3}E_{n}^{\prime }({h_{n}}^{3}-{h_{n-1}}^{3})$$

where *h_n_* is the distance from the top of the *n*th layer to the neutral axis (*z*_NA_). The plate modulus (*E*’*_n_* = *E_n_*/(1 − *υ_n_*^2^)) where *E_n_* and *υ_n_* are the layer’s Young modulus and Poisson ratio.

The membrane of the conventional pMUT is fully covered with a piezoelectric thin film. In this study, however, the PZT thin film was partially etched except for the membrane and the interconnection area to the topside electrode. This structure leads to an increase of the residual stress in the piezoelectric thin film and eventually enlarges the displacement of the membrane in comparison to the conventional pMUT without the etching. A higher sensitivity was obtained for the receiving mode for the same reason [29,30]. The covering ratio of the top electrode to the membrane diameter was set to approximately 75% in this study [31,32]. The pitch of the linear array was about 100 μm.

**Figure 2:**
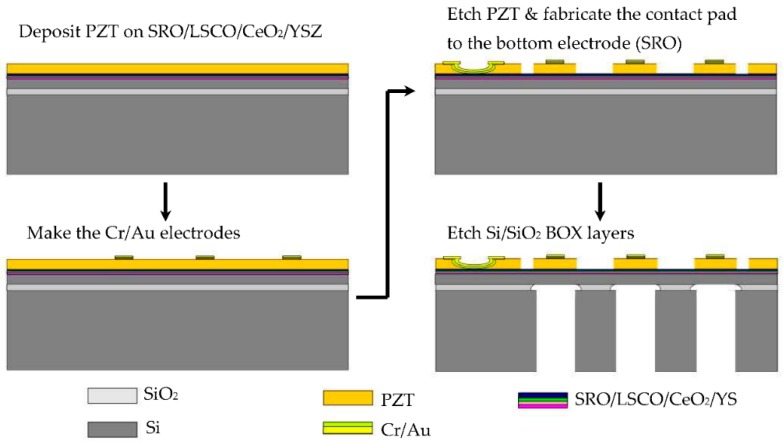
Process chart of pMUT array based on PZT thin film.

The process chart is illustrated in Figure 2. At first, a 1.7-µm-thick PZT was deposited on a SOI substrate in the same manner as above. Then, Au/Cr top electrodes with 100 nm/20 nm thickness were fabricated. In addition, the Au/Cr layers were also sputtered on SRO electrode for the wire bonding. The PZT thin film was patterned by wet etching in acid solution containing HF, HNO_3_, NH_4_F, CH_3_COOH and H_2_O (40:93:3:34:30 in volume). After that, the Si handle layer was etched by deep reactive ion etching. Finally, the SiO_2_ buried oxide (BOX) layer was etched with a HF solution. 

## 3. Results and Discussion

### 3.1. Characterization of PZT Fibered-Epitaxial Thin Film on Si

**Figure 3:**
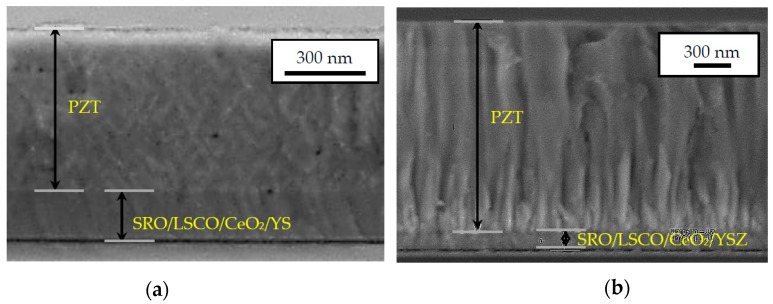
Typical SEM cross-section images of (**a**) monocrystalline and (**b**) fibered-epitaxial thin films.

Figure 3 shows a cross-sectional view of the PZT monocrystalline and fibered-epitaxial thin films. Ultrahigh resolution analysis scanning electron microscope (SEM) SU-70 (Hitachi, Tokyo, Japan) was used. No significant grain boundaries were observed in the former, while the fibered structures with grain boundaries were observed in the latter. 

**Figure 4:**
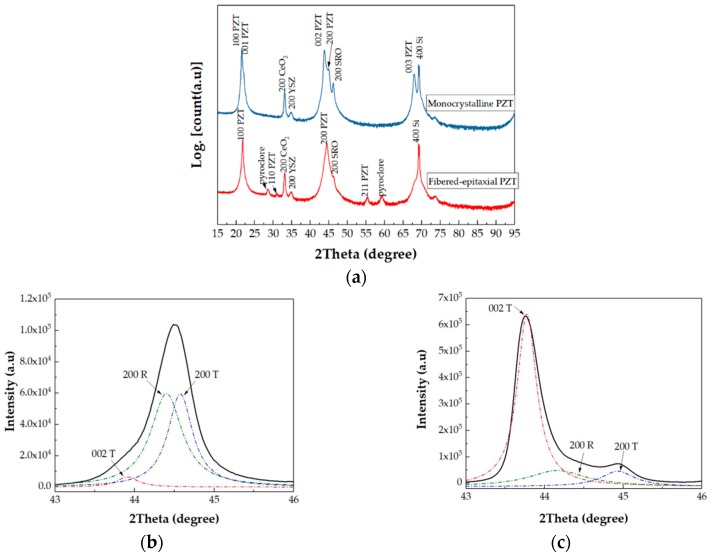
(**a**) Typical X-ray diffraction (XRD) patterns of the monocrystalline and fibered-epitaxial PZT films. Analysis (200)/(002) peaks of (**b**) fibered-epitaxial and (**c**) monocrystalline films by Lorentz-function-curve fitting.

Figure 4a shows the X-ray diffraction (XRD) pattern of each specimen by using Bruker D8 DISCOVER (Bruker, Karlsruhe, Germany). In the monocrystalline thin film, the *c*-axis orientation was dominant. On the other hand, the (100)/(001) orientations were also dominant in the fibered-epitaxial thin film, although the small peaks corresponding to the other orientations were seen. The phase component in the fibered-epitaxial thin film was evaluated by analyzing the (200)/(002) peak in detail, as shown in Figure 4b. These diffraction peaks were well fitted to the three Lorentz-function curves corresponding to the (002) tetragonal, (200) rhombohedral, and (200) tetragonal phases. The content of the tetragonal phase was 52%, which was calculated from the peak-intensity ratio of the [(200) + (002)] tetragonal phase to the sum intensity of {[(200) + (002)] tetragonal + (200) rhombohedral}. The amount of the tetragonal phase was close to the rhombohedral phase. On the other hand, the monocrystalline PZT film included a slight amount of rhombohedral phase in in spite of the fact that these films were prepared from the same sputter target, as shown in Figure 4c.

**Figure 5:**
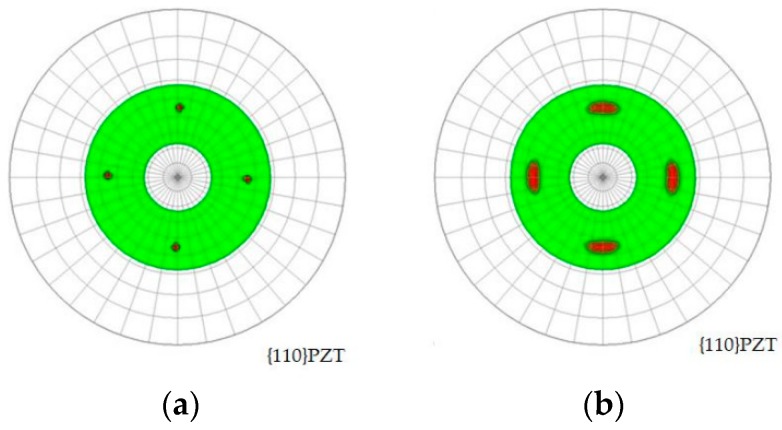
In-plane epitaxial relationships of (**a**) monocrystalline and (**b**) fibered-epitaxial films by pole figures.

The in-plane epitaxial relationships of both films were investigated by obtaining the pole figures, as shown in Figure 5. Only four diffraction spots were observed for both specimens. This result verifies that (100)/(001) crystalline lattice was epitaxially grown with four-fold symmetry. Note that the conventional polycrystalline thin film with random orientation in the in-plane direction should exhibit a circle-like pole figure. The spot size of the fibered-epitaxial thin film was larger than that of the monocrystalline thin film because of its lower monocrystallinity and the existence of multi-domains or phases. Thus, the fibered-epitaxial thin film as an intermediate-categorized thin film between monocrystal and polycrystal was fabricated on Si successfully.

**Figure 6:**
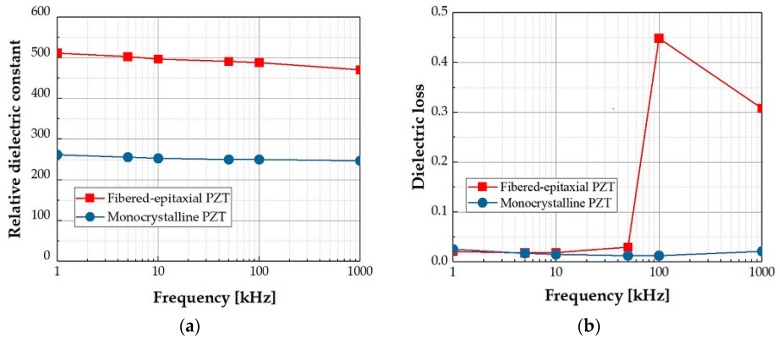
Dependencies of (**a**) the relative dielectric constant and (**b**) dielectric loss on the measurement frequency.

Then, the relative dielectric constant and dielectric loss (tan *δ*) were measured with an LCR meter (IM3536, HIOKI E.E. Corporation, Nagano, Japan) by applying an alternate-current voltage with an amplitude of 0.2 V to a Pt-PZT-SRO sandwich structure at room temperature. In this study, all experiments were performed for the as-prepared thin film without poling treatment. Figure 6a plots the dependency of the relative dielectric constant on the measurement frequency. The dielectric constants of the monocrystalline and fibered-epitaxial thin films were approximately 250 and 500, respectively. Although the value of the fibered-epitaxial thin film was larger, it was still much smaller than those of the conventional polycrystalline thin films near the MPB compositions, as mentioned above. This fibered-epitaxial thin film had an intermediate dielectric property between the single crystal and polycrystal. Our result was similar to the prior reports on (100)/(001)-oriented Pb(Zr_0.35_Ti_0.65_)O_3_ epitaxial thin films with three in-plane variants [23,24,25]. The dielectric constant of the fibered-epitaxial thin film decreased slightly with increasing frequency in comparison with the monocrystalline thin film. This might be caused by a reduction of the extrinsic contributions increasing the dielectric constant, e.g., charge traps in the grain boundaries [21,22]. Figure 6b plots the dependency of tan *δ* on the measurement frequency. Both films exhibited about 0.02 as tan *δ* in the low frequency range less than 50 kHz. However, the value of only the fibered-epitaxial thin film jumped up in the higher frequency range. The phenomenon might be related to the existence of the grains or the mixture of the domains. The interaction between the domain walls perhaps induced the fluctuation of tan *δ* [33,34].

In addition, the residual stresses of the fibered-epitaxial and monocrystalline thin films were also compared. The tensile stresses in the fibered-epitaxial and monocrystalline thin films measured approximately 200 MPa and 250 MPa, respectively. The increase of the *a*-domain ratio and the existence of the grain boundary in the in-plane direction probably relaxed the residual stress for the former.

### 3.2. Actuation Performance and Mechanical Toughness of pMUT Based on PZT Fibered-Epitaxial Thin Film 

#### 3.2.1. pMUT Using Partially-Etched Structure

**Figure 7:**
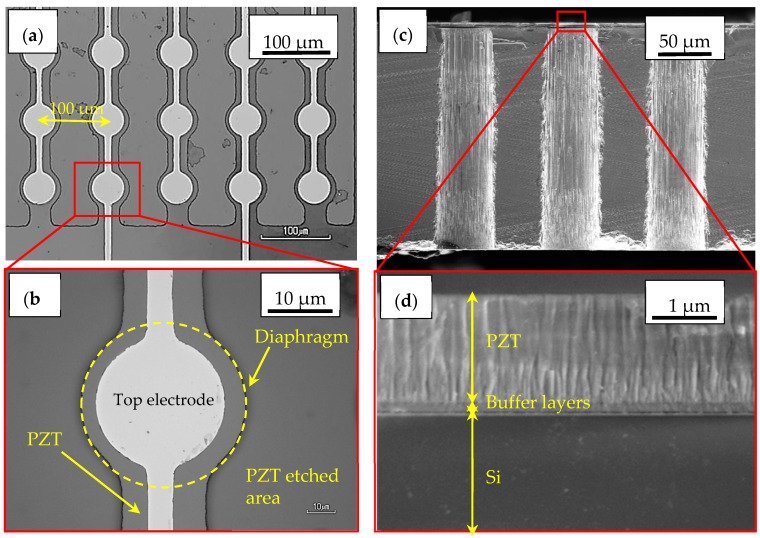
Optical images of (**a**) pMUT linear array and (**b**) single element, respectively; (**c**,**d**) Cross-sectional SEM images of the array and fibered-epitaxial thin film on the membrane.

The pMUT linear arrays were successfully fabricated, as shown in Figure 7a. As mentioned above, two types of the device were fabricated. One was mounted on the blanket PZT layer. The other was mounted with the PZT layer partially-etched except for the membrane area. Figure 7b shows the pMUT array with the partially-etched PZT thin film. The misalignment between the top electrode and hole was less than 3 μm. As shown in Figure 7d, the PZT fibered-epitaxial thin film shows a dense columnar structure.

**Figure 8:**
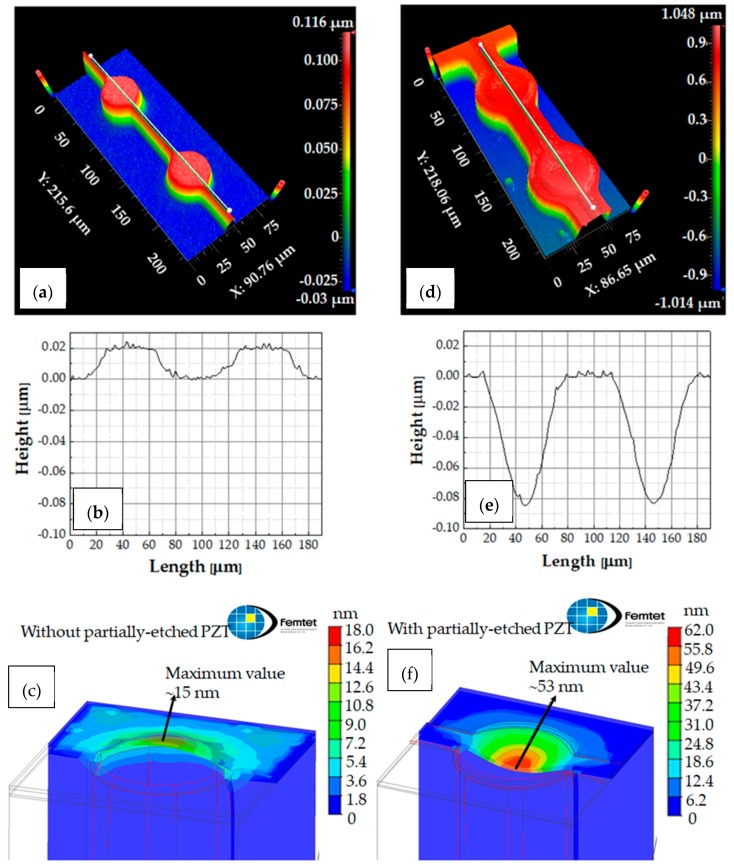
Three-dimensional images of the surfaces for pMUTs (**a**) without and (**d**) with the partial etching of PZT. Profiles along (**b**) A-A’ in (**a**) and (**e**) B-B’ line in (**b**), respectively. Simulation results of the membranes’ deformations (**c**) without and (**f**) with the partial etching of PZT, respectively.

The curvatures of the membrane for both devices were observed using a white light interference meter (NewView™ 9000 3D Optical Surface Profiler, ZYGO, Middlefield, CT, USA). Figure 8a,d are the three-dimensional images of the surfaces for the pMUTs without and with etching PZT, respectively. Figure 8b,e are the profiles along A-A’ and B-B’ line in Figure 8a,d, respectively. The membrane fully covered with the PZT thin film was almost flat or buckled slightly due to influence from the compressively-stressed SiO_2_ BOX layer. On the other hand, the membrane with the PZT etching buckled down. This was probably caused by the stress-release effect of the PZT thin film on the membrane due to the etching in the outer area. This hypothesis was supported by a simulation result using the finite element method (FEM), as shown in Figure 8c,f. The membrane was assumed to be composed of only PZT and Si elastic plate to simplify the simulation. The PZT and BOX layers were considered to have a tensile stress of +200 MPa, which was the measured value as described above, and a compressive stress of −300 MPa, respectively. Table 1 lists the parameters of the device structure and material properties for the simulation. As seen in these figures, the simulation results well matched the observed phenomena.

#### 3.2.2. Characterization of the PZT Fibered-Epitaxial Thin Film via the Actuation Experiments of the Fabricated pMUT

**Figure 9:**
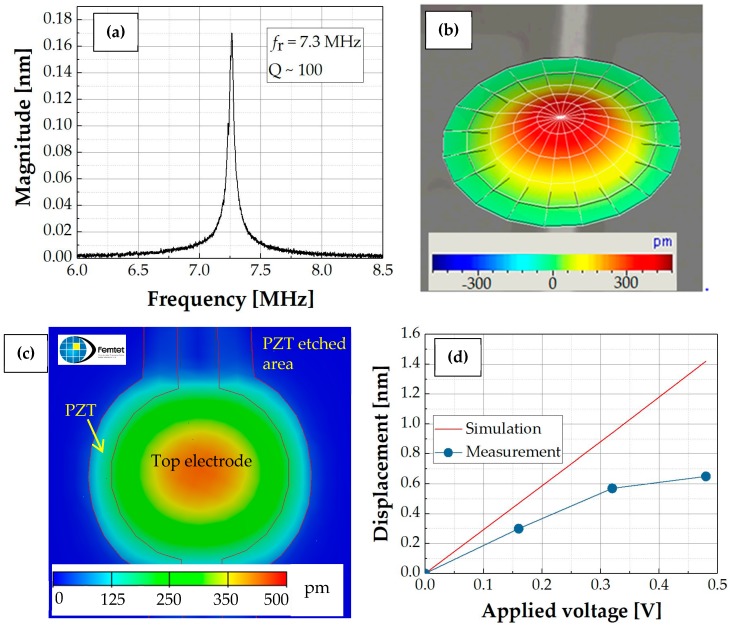
(**a**,**b**) Laser Doppler vibrometer (LDV) measurement results for 60 μm diameter of PZT PMUT by applying 0.16 V of driving voltage; (**c**) Simulation result on pMUT with the same geometry and operating condition as the real device; (**d**) Comparison between the simulation and measurement results.

The pMUT with the partially-etched PZT fibered-epitaxial thin film was characterized in air by a laser-Doppler vibrometer (MSA-500 micro system analyzer, Polytec GmbH, Waldbronn, Germany). The resonant frequency and mechanical quality factor were 7.3 MHz and 100, respectively, as shown in Figure 9a. The shift of the resonant frequency from the calculated value was probably caused by a fabrication error and/or the stress of the membrane. The maximum displacement was observed at the center of the membrane, as shown in the displacement mapping image (Figure 9b). This suggests that no significant misalignment occurred between the topside electrode and membrane. 

In order to evaluate the piezoelectricity (*e*_31,*f*_) of the PZT fibered-epitaxial thin film, we simulated the displacement of the membrane by FEM, as shown in Figure 9c. This simulation adapted the material parameters of P-5A for the piezoelectric transducer thin film, which is an industry type 5A (Navy type II) piezoelectric ceramic [35], listed in Table 2. For the simulation, the pattern of the Au topside electrode with a thickness of 0.15 µm was considered, while the buffer layers were ignored. The *e*_31,*f*_ of P-5A were calculated to be −16 C/m^2^ from the following expression [36]

(4) $$e_{31,f}=e_{31}-\frac{C_{13}}{C_{33}}\;e_{33}$$

where *e* and *c* correspond to the piezoelectric and stiffness coefficients, respectively. Figure 9d plots the dependencies of the static displacements on the driving voltages for the simulation and measurement results. The prototyped pMUT showed a non-linearity above about 0.3 *V_p_*. This non-linearity was probably caused by the stiffening effect of the stiffening diaphragm [37]. As seen in this graph, the measured displacement of the measured result was two thirds of the simulated result in the linear region. This means that the actual *e*_31,*f*_ of the PZT fibered-epitaxial thin film should also be two thirds of −16 C/m^2^, i.e., −10–−11 C/m^2^ because the relationship of the static displacement and *e*_31,*f*_ are proportional, which is supported by the following expression [38]

(5) $$d_{s}=-r^{2}\frac{e_{31,f}\left(t_{\mathrm{Si}}+t_{\mathrm{buffer}}+\frac{t_{\mathrm{PZT}}}{2}-z_{\mathrm{PZT}}\right)\cdot I_{p}\left(r\right)}{D\cdot I_{d}}$$

where *t*_si_, *t*_buffer_ and *t*_PZT_ are the thicknesses of Si elastic plate, the buffer layer and the PZT thin film, respectively. *z*_PZT_ is the distance from the middle of the PZT film to the neutral axis. The flexural rigidity *D* for the membrane is defined as Equation (3). *I*_p_(*r*) and *I*_d_ are integrals related to the piezoelectric bending moment and modal stiffness of the pMUT.

The estimated value of *e*_31,*f*_ was equal or higher than those of conventional PZT polycrystalline thin films near the MPB composition without poling [31,39,40,41]. Therefore, the FOM of the fibered epitaxial thin film for pMUTs, (*e*_31,*f*_)^2^/*ε_r_*_33_*ε*_0_ were calculated to be 22–27 GPa. This value is superior to the conventional PZT polycrystalline thin films (6–18 GPa [42]). Moreover, no specific poling treatment was performed in this study. A poling treatment will improve *e*_31,*f*_ thanks to an increase of the volume fraction of the (001) orientation and domain walls [43], resulting in a larger FOM than the as-prepared thin film.

**Figure 10:**
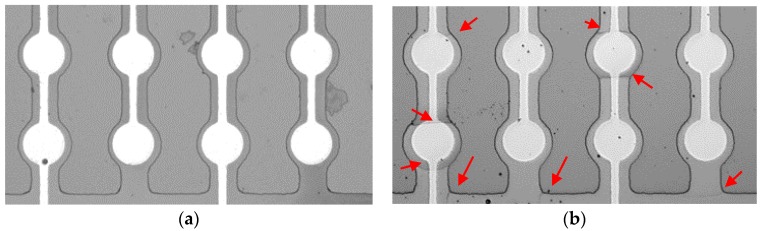
Mechanical behaviors of pMUTs with partial-etching using (**a**) fibered epitaxial and (**b**) monocrystalline films.

Finally, we observed the crack generation behavior of the pMUTs with the partially-etched fibered-epitaxial and monocrystalline thin films. Neither mechanical breaks nor crack generation were observed in the fibered-epitaxial thin film even after the above-mentioned actuation test, as shown in Figure 10a. By contrast, many cracks were generated in the monocrystalline thin film, as shown in Figure 10b. They often appeared at the corners of the etched area corresponding to stress-concentrated points. This result is reasonable considering the failure mechanism of a brittle material, and implies that the stress-concentrated points (such as the corner or the rough-edged part) should be eliminated for use of the monocrystalline thin film. The existence of the grain boundaries in the in-plane direction perhaps inhibits the crack propagations in the thin film. Thus, it was demonstrated that the fibered-epitaxial thin film has a higher mechanical toughness than the monocrystalline one. The mechanical toughness and flexibility of design are attractive in terms of the reliability of the device and practical applications.

Therefore, this study successfully demonstrated that the fibered-epitaxial PZT thin film had unique well-balanced properties; a reasonably-high FOM for pMUT thanks to the smaller dielectric constant and good mechanical toughness.

## 4. Conclusions

In this paper, a fibered-epitaxial PZT thin film was fabricated on Si and characterized for the application to pMUT. The XRD analytical results proved that this fibered-epitaxial thin film had an intermediate-categorized crystalline structure between monocrystal and polycrystal. The (200) tetragonal and (200) rhombohedral phases were dominant in this film. The dielectric constant measured approximately 500, which was relatively low compared to other MPB-composition PZT polycrystals. This may be thanks to the partial epitaxial structures. The piezoelectric constant *e*_31*,f*_ of the thin film without poling treatment was estimated from the actuation tests of the prototyped pMUT to be −10–−11 C/m^2^ by prototyping the actual pMUT and measuring the actuation behaviors. The resultant FOM for pMUT ranged from 22 to 27 GPa. The value was already larger than those of the polycrystalline thin films, and will be improved by the poling treatment. The mechanical toughness of this fibered epitaxial thin film was also superior to the monocrystalline thin film. We believe that such a unique epitaxial thin film on Si has the potential to provide a well-balanced pMUT, satisfying a high signal-to-noise ratio, mechanical toughness and design flexibility.

## Figures and Tables

**Table 1:** Material properties and thicknesses used in the residual stress simulation.

Material	Young Modulus *E* (GPa)	Residual Stress *σ* (MPa)	Poisson’s Ratio	Initial Strain ($\varepsilon =\frac{\sigma }{E}$)	Thickness (µm)
SiO_2_	73.1	−300	0.17	−0.0040	1.0
Si-device layer	180	-	0.3	0	2.5
PZT	76	+200	0.3	+0.0026	1.7

**Table 2:** Fundamental properties of P-5A [35] and silicon materials in this simulation.

Mechanical Properties	PZT-5A	Si
Stiffness coefficient (GPa)		
*c*_11_	121	165.7
*c*_12_	75.4	63.9
*c*_13_	75.2	63.9
*c*_33_	111	165.7
*c*_44_	21.1	79.6
Piezoelectric coefficient (C/m^2^)		
*e*_31_	−5.4	-
*e*_33_	15.8	-
*e*_15_	12.3	-
Relative permittivity		
*ε_r_*_11_	915	11.8
*ε_r_*_33_	829	11.8
